# Antibody targeting of Cathepsin S induces antibody-dependent cellular cytotoxicity

**DOI:** 10.1186/1476-4598-10-147

**Published:** 2011-12-14

**Authors:** Hang Fai Kwok, Richard J Buick, Diana Kuehn, Julie A Gormley, Declan Doherty, Thomas J Jaquin, Angela McClurg, Claire Ward, Teresa Byrne, Jacob Jaworski, Ka Lai Leung, Philip Snoddy, Christine McAnally, Roberta E Burden, Breena Gray, Jenny Lowry, Isabelle Sermadiras, Natalia Gruszka, Nigel Courtenay-Luck, Adrien Kissenpfennig, Christopher J Scott, James A Johnston, Shane A Olwill

**Affiliations:** 1 Fusion Antibodies Ltd., Springbank Ind. Est. Belfast, BT17 0QL, Northern Ireland; 2 Molecular Therapeutics, School of Pharmacy, Queen's University Belfast, BT9 7BL, Northern Ireland; 3 Centre for Infection and Immunity, School of Medicine, Dentistry and Biomedical Sciences, Queen's University Belfast, BT9 7BL, Northern Ireland

**Keywords:** Cathepsin S, ADCC, antibody, protease, microenvironment

## Abstract

**Background:**

Proteolytic enzymes have been implicated in driving tumor progression by means of their cancer cell microenvironment activity where they promote proliferation, differentiation, apoptosis, migration, and invasion. Therapeutic strategies have focused on attenuating their activity using small molecule inhibitors, but the association of proteases with the cell surface during cancer progression opens up the possibility of targeting these using antibody dependent cellular cytotoxicity (ADCC). Cathepsin S is a lysosomal cysteine protease that promotes the growth and invasion of tumour and endothelial cells during cancer progression. Our analysis of colorectal cancer patient biopsies shows that cathepsin S associates with the cell membrane indicating a potential for ADCC targeting.

**Results:**

Here we report the cell surface characterization of cathepsin S and the development of a humanized antibody (Fsn0503h) with immune effector function and a stable *in vivo *half-life of 274 hours. Cathepsin S is expressed on the surface of tumor cells representative of colorectal and pancreatic cancer (23%-79% positive expression). Furthermore the binding of Fsn0503h to surface associated cathepsin S results in natural killer (NK) cell targeted tumor killing. In a colorectal cancer model Fsn0503h elicits a 22% cytotoxic effect.

**Conclusions:**

This data highlights the potential to target cell surface associated enzymes, such as cathepsin S, as therapeutic targets using antibodies capable of elicitingADCC in tumor cells.

## Background

Proteases regulate a number of pathways relevant to cancer biology, including proliferation, differentiation, apoptosis, migration, and invasion [1,2]. In the last decade, it has become increasingly evident that tumor cells create a pericellular microenvironment where molecules such as metalloproteinases, cysteine proteases and serine proteases interact to form a pro-tumorigenic proteolytic network [2,3]. Indeed the establishment of a causal relationship between enhanced activity or expression of proteases and tumor progression (e.g. through extracellular matrix remodelling) has promoted the development of many small molecule inhibitors as anticancer therapeutics. However clinical trials with many of these agents have been disappointing due to their off target effects coupled with poor bioavailability, leading drug developers to consider the use of biologic inhibitors (antibodies or peptides) [1,4,5]. There is an increasing body of evidence suggesting that proteases involved in cancer microenvironment which are normally found within intracellular compartments often relocate during tumor progression, resulting in secretion and association with binding partners on the tumor cell surface [6-9].

Cathepsin S is one of a family of eleven lysosome cysteine proteases normally restricted to the lysosomes of professional antigen presenting cells where it mediates cleavage of the invariant chain (li) from MHC class II complexes prior to antigen loading for presentation [10-12]. In cancer, cathepsin S is translocated from its normal intracellular lysosomal compartment into the extracellular milieu [13,14]. Reports have shown that cathepsin S is stable at neutral pH and is potently elastin- and collagenolytic, promoting extracellular matrix remodelling, tumor growth and invasion in the tumor microenvironment [15,16]. Enhanced cathepsin S expression and activity have been detected in several human cancers (glioma, breast, prostate, colorectal and pancreatic) with *in vivo *mouse models supporting its role in tumorigenesis [17-21]. The association of cathepsin S with colorectal cancer progression has been recently highlighted where it was shown to be a prognostic indicator [22]. A number of groups have studied the mechanistic role of cathepsin S in cancer using *in vitro *and *in vivo *models [18,21].

The potential of cathepsin S as a novel cancer target amenable to antibody mediated therapy has been examined using a murine anti-cathepsin S monoclonal antibody (Fsn0503) which is capable of blocking tumor cell invasion, endothelial tube formation and microvascular sprouting during angiogenesis [23,24]. While previous reports had suggested that cathepsin S is found either in the lysosomal lumen or secreted into the ECM, our analysis of colorectal cancer patient biopsies and cancer cell lines show that it is also associated with the cell membrane indicating a potential for antibody dependant cellular cytotoxicity (ADCC) targeting.

ADCC relies on a mechanism of Fc effector domain recruitment of immune cells (e.g. Natural Killer) to tumor cells with surface bound antibody. Advances in recombinant antibody engineering facilitate the introduction of immune effector function for those antibodies which target cell surface antigens [25,26].

In the present study, we show that cathepsin S is on the surface of tumour cells and that this localization can be exploited with a fully human IgG1 version of Fsn0503 (Fsn0503h) to induce ADCC, demonstrating the clinical potential of the engineered cathepsin S specific human antibody Fsn0503h.

## Results

### Cathepsin S is expressed on the surface of Colorectal Cancer (CRC) tumor cells

**Figure 1:**
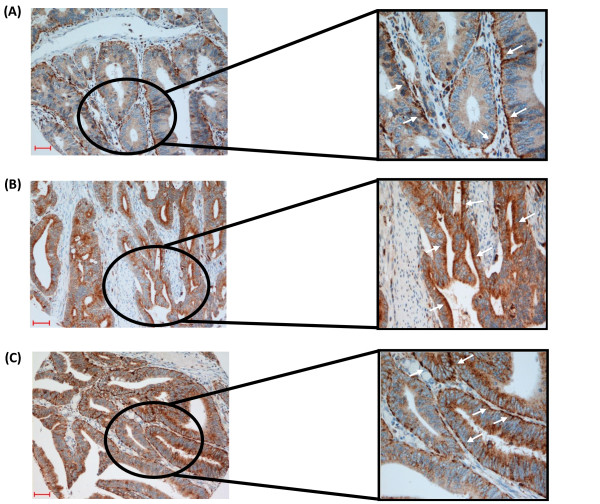
**Representative images (A, B and C)**: Polarised cathepsin S expression patterns in colorectal cancer patient biopsies. Cathepsin S-specific staining is brown (indicated with arrow heads) and nuclear counterstaining is blue. (Scale bars - 100 μm).

The prevalence of cathepsin S (>95% patients) in CRC was recently demonstrated in a large-scale IHC study spanning three cohorts of patient samples (n = 561) [22]. In addition to the relationship of Cathepsin S levels with the disease, a distinct polarization to either the basal or apical epithelial membrane was also observed in 40% of cases, as shown in Figure 1, which is suggestive of cell surface localization and potential secretion of the protease into the tumor microenvironment.

**Figure 2:**
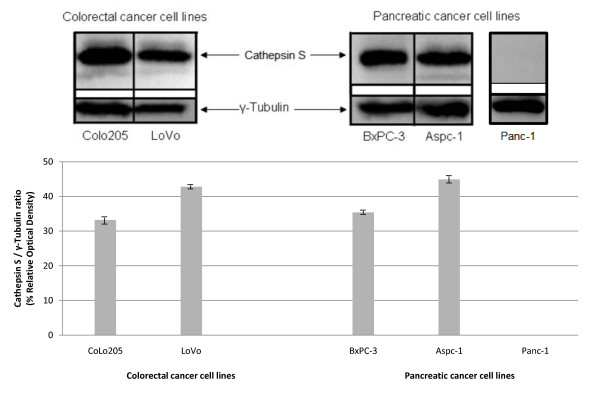
**Western blot analysis of cathepsin S on different cancer cell lines**: Colo205, LoVo, BxPC-3, Aspc-1 and Panc-1. Density of the bands (Cathepsin S/γ-Tubulin ratio) is represented as % of relative optical density units.

**Figure 3:**
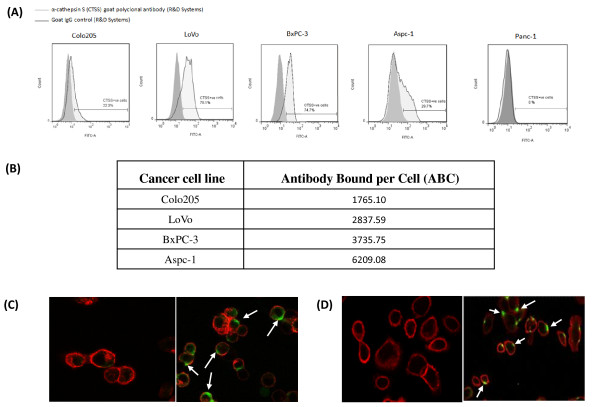
**Cathepsin S surface expression quantification on cancer cell lines**. (A) Cathepsin S surface expression on Colo205, LoVo, Bx-PC3, Aspc-1 and Panc-1 by flow cytometry. (B) QuantiBRITE PE beads fluorescence quantification of cathepsin S expressed on the surface of cathepsin S-positive cell lines (Colo205, LoVo, BxPC-3 and Aspc-1). (C, D) Confocal microscope images of LoVo (C) and Aspc-1 (D) cell lines show human tumor expression of cathepsin S on the cell surface (Colo205, BxPC-3 and Panc-1 shown in supplementary figures): Left panels show isotype control while right panels show cathepsin S specific antibody (green) binding to the cell surface, indicated with arrow heads.

We next examined the expression of cathepsin S on a panel of cancer cell lines (Colo205, LoVo, BxPC-3, Aspc-1 and Panc-1) by western blot. A clear band was observed at approximately 28 kDa for all the cell lines, except Panc-1 which was found to be cathepsin S negative (included as a negative control), (Figure 2). The results confirmed expression of the protease in Colo205, LoVo, BxPC-3 an Aspc-1 cell lines and we also demonstrated its secretion into the growth media (data not shown). As we postulated that cathepsin S is translocated from its intracellular compartment to associate with the tumor cell surface prior to secretion, we examined the cell surface expression of cathepsin S across the panel of cell lines by flow cytomtery. A significant amount of cell surface cathepsin S was detected in our panel of cancer cell lines. A higher percentage of LoVo and BxPC3 cells stained positive for surface cathepsin S (79.6% and 74.4% respectively) compared to the Colo205 and Aspc-1 cells (22.3% and 29.7% respectively) (Figure 3A). To perform a comparative quantification of cell surface associated cathepsin S across the cell lines we used the QuantiBRITE PE beads flow cytometry system. Using a 1:1 target to antibody ratio we observed a similar level of cathepsin S expression on all cell lines. The results demonstrated that there was between 1765-6209 antibodies bound per cell (ABC) indicating a similar range of cathepsin S antigen density expressed on each cancer type assessed (Figure 3B).

To further assess the translocation of cathepsin S from intracellular compartments to the cell surface, cancer cell lines (Colo205, LoVo, BxPC-3 and Aspc-1) were analysed by confocal microscopy. In all cell lines, intense cathepsin S staining co-localizes with actin at focal points on the cell membrane mirroring the expression pattern observed in patient biopsy samples (Figure 3C &3D). This suggests a concentrated localization of cathepsin S at specific areas of the cell surface. This expression pattern of localised regions of high antigen density is observed in a subset of cells potentially indicating tight regulation of surface expression. In some cases, cathepsin S expression can be observed in protrusions of the cell membrane, potentially areas of exocytosis.

### Humanization and characterization of Fsn0503

**Figure 4:**
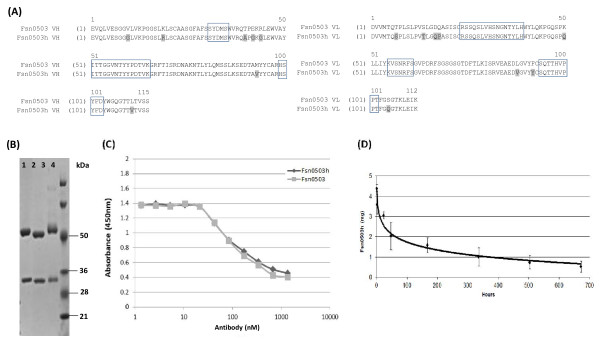
**(A) Alignment of Fsn0503 variable domains and designed Fsn0503h human variable domains**. Amino acid residues mutated from the murine sequence are highlighted. CDRs are boxed. (B) 4-20% SDS gel analysis showed the highly purified cathepsin S antibodies: Lane 1 -- Fsn0503h; Lane 2 -- Fsn0503; Lane 3 - Human isotype antibody control; Lane 4 -- SeeBlue plus marker. (C) Compeptitive immunoreactivity profile: Fsn0503h & Fsn0503 was allowed to compete with a biotin-labeled Fsn0503 (120 nM) for binding to recombinant Fsn0503 mature antigen (40 nM). (D) Pharmacokinetic analysis of Fsn0503h in rats. Following a single i.v. administration of Fsn0503h using a bi-exponential model (linear scale), it showed that the initial half-life value of Fsn0503h was 14.8 hours with a terminal half-life of 274 hours (approximately 11.4 days).

To facilitate future clinical development the murine antibody, Fsn0503, was humanized using recombinant antibody engineering. The Fsn0503h variable domain sequences are shown in alignment with the parental Fsn0503 murine variable domain sequences in Figure 4A with all constant determining regions (CDR's) conserved. The constant domains of the antibody (isotype) were selected as human IgG1 in order to confer ADCC activity to the human version of the antibody. A stable pool of NSO1 cells expressing Fsn0503h was selected using the DHFR inhibitor MTX (200 nM). A single high yield cell line was then isolated by limiting dilution for downstream production. Purified antibodies were analysed by SDS-PAGE (Figure 4B) and tested in a competition ELISA for binding to their target antigen, cathepsin S, against biotinylated murine anti-cathepsin S (Figure 4C). The Fsn0503h heavy chain and light chain can be clearly seen on the SDS-PAGE gel at 50-and 32-kDa respectively. As expected, they are slightly different in size to their murine Fsn0503 equivalent chains due to the differing size of the constant domains. The competition ELISA (Figure 4C) demonstrates that the Fsn0503h antibody displays a similar binding profile to the parental Fsn0503 antibody, indicating no change in affinity during the humanization process which is further supported by similar affinities and off rates when assessed by biacore and comparable *in vitro *efficacy (data not shown). In order to evaluate the potential serum half-life of Fsn0503h and blood clearance rates a pharmacokinetic study was performed in SD rats. The PK profile for each individual animal was analysed as well as the analysis of pooled serum samples for each time point. Both methods of analysis gave similar PK profiles. Analysis of data using a bi-exponential model gives an initial half-life value of 14.8 hours with a terminal half-life of 274 hours (Figure 4D). Taken together the results demonstrate the successful engineering of Fsn0503h into an experimental therapeutic with favorable pharmacokinetic properties.

### ADCC of Fsn0503h in colon and pancreatic carcinoma cell line

**Figure 5:**
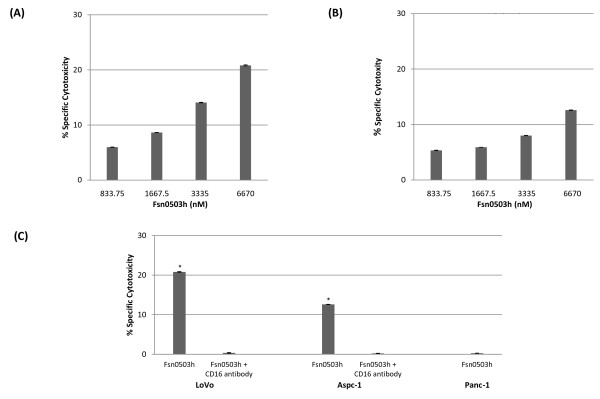
**Fsn0503h specifically induces antibody-dependent cellular cytotoxic (ADCC) killing of tumor cells through a CD16 dependent mechanism**. (A, B) The dose dependent ADCC activity of Fsn0503h in (A) LoVo and (B) Aspc-1 cell lines. LoVo/Aspc-1 cells were incubated with human peripheral blood mononuclear cells (PBMCs) obtained from healthy donors which were stimulated with IL-2 (100 U/mL) at an E/T ratio of 40/1 in the presence of various concentrations of Fsn0503h. (C) ADCC activity of Fsn0503h (6670 nM) in LoVo and Aspc-1 cell line was blocked in the presence of CD16 neutralising antibody (6670 nM). The cathepsin S-negative cell line (Panc-1) was used as a negative control. *P < 0.01 (paired *t*-test) for Fsn0503h versus Fsn0503h+CD16 antibody.

ADCC relies on the mechanism of cell-mediated immunity whereby an effector cell actively lyses a tumor cell which has been bound by a specific antibody. To determine the ADCC potential of our humanized antibody, Fsn0503h, we evaluated its efficacy to kill LoVo and Aspc-1 cancer cell lines by ADCC using an LDH release assay. Fsn0503h was capable of mediating cytotoxic killing of target cancer cells in a dose-dependent manner (from 883.75 nM to 6670 nM) when assayed with isolated human donor PBMCs. The dose-dependent cytotoxic effect of Fsn0503h on LoVo and Aspc-1 cell lines had a maximal efficacy of approximately 22% and 13% respectively (Figure 5A). In order to demonstrate which Fc receptor is engaged in PBMC-mediated killing by Fsn0503h the ADCC experiments were also performed in the presence of a specific FcγRIII blocking antibody (CD16). Killing by isolated PBMCs was effectively neutralised by the presence of CD16 antibody (6670 nM) demonstrating the required involvement of FcγRIII on effector cells (e.g. natural killer (NK)cells) in Fsn0503h dependent ADCC (Figure 5B). The cathepsin S-negative cell line Panc-1 was also used as a control to show the ADCC efficacy of Fsn0503h is due to the specific binding of cathepsin S.

## Discussion

Here we demonstrate expression of cathepsin S on the surface of pancreatic and colon carcinoma cells. In addition, we reveal how cell surface associated cathepsin S can be targeted to mediate ADCC by a fully humanized anti-cathepsin S antibody, Fsn0503h. To the best of our knowledge this is the first example of a protease or surface enzyme being targeted to elicit ADCC.

Cathepsin S is a cysteine protease that plays a key role in invasion, angiogenesis and metastasis during tumor progression [21,27-29]. In normal conditions, cathepsin S has limited tissue distribution and is found primarily in lysosomal compartments of professional antigen presenting cells; however it is up-regulated and secreted into the microenvironment during tumor development [11,21]. Based on our findings, we postulate that during tumorigenesis, a proportion of secreted cathepsin S associates with the tumour cell surface. Cathepsin B has previously been shown to localize to the cell surface [30]. Under normal circumstances cathepsin B, a house-keeping enzyme, is located in perinuclear lysosomes but in cancer it is secreted and relocalized to the plasma membrane. While the exact mechanisms underlying intracellular enzyme shuttling of cathepsins remain to be elucidated it is thought that mannose-6-phosphate receptors (MPR) may play a role albeit via discrete pathways for individual family members [31]. Under certain conditions, including reduced pH, lysosomes may fuse with the plasma membrane, thus becoming secretory organelles, which facilitate association with the cell surface [32]. This membrane association is supported by our flow cytometry findings which show on average 23-79% of Colo205, LoVo, BxPC-3 and ASPC1 cells express cell surface cathepsin S. IHC analysis of patient biopsies further demonstrate cell surface localization of cathepsin S where staining is characterized by a distinct polarization to either the basal or apical epithelial membrane. Confocal analysis of cancer cell line models also support this association as cathepsin S was observed to localize to the cell surface membrane with intense staining restricted to specific regions. A pattern of regional expression has also been reported for other proteases involved in tumorigenesis which have been shown to congregate in caveolae. Caveolae have multiple proteolytic factors (e.g. cathepsins, annexins, uPA, MMP's) which represent concentrated membrane sub-domains (protease pools) that facilitate organized cell surface proteolysis [33]. As well as facilitating co-operation between different proteases the concentration of cathepsin S in focal micro-domains, results in regions of high antigen density, which can beused to induce ADCC.

As discussed above, Fsn0503 is a murine IgG1κ antibody, shown to have anti-tumor efficacy in xenograft models through inhibition of cathepsin S mature protein. The murine IgG1 isotype has negligible ADCC potential and has the added drawback that if administered to patients undergoing immunotherapy may result in a strong human anti-mouse antibody (HAMA) response with rapid clearance. To make the antibody suitable for clinical utility we have developed a fully human version. The majority of therapeutic antibodies are human IgG1 isotypes (e.g. Herceptin and Cetumximab), particularly in oncology as it facilitates the introduction of additional effector function. IgG1 allows engagement of immune effector cells through binding of Fc receptors (FcγR) expressed on NK cells, macrophages and polymorphonuclear leukocytes with the NK population identified as the principal instigator of ADCC [34-36]. IgG4 isotype antibodies such as natalizumab (Tysabri) are more commonly used in applications where cell killing is not the objective, such as multiple sclerosis or rheumatoid arthritis. Based on our findings that cathepsin S is associated to the surface of the tumour cells we engineered a human antibody with ADCC functionality. Through the use of a specific human IgG1 framework the recombinant antibody, Fsn0503h with an *in vivo *half-life of approximately 11.4 days (274 hours), has the potential to recruit NK cells via the FcγRIIIa (CD16a)and to actively kill tumor cells in patients [35]. In order to evaluate the ADCC effector function of Fsn0503h we performed *ex vivo *activity assays using healthy donor peripheral blood mononuclear cells as it is difficult to obtain meaningful data from current *in vivo *murine models. Human IgG1 antibodies have much reduced ADCC activity in mouse xenograft models due to differing species immune cell recognition patterns [37,38]. We chose to assess the effector function of our human antibody using a well characterized LDH release assay and demonstrated that Fsn0503h is capable of eliciting greater than 20% specific cytotoxic cell kill in the LoVo colorectal cancer model. Furthermore with the use of an anti-CD16 neutralising antibody we have shown that the ADCC functionality of Fsn0503h works through the recruitment of CD16 positive immune cells such as human NK cells to induce tumour cell death.

Currently twenty-two therapeutic antibodies have been approved by the US Food and Drug Administration (FDA), at least eleven of which are for the treatment of cancer [39,40]. While antibodies such as rituximab have proven very efficacious for the treatment of leukaemia the clinical impact of drugs such as cetuximab (anti-EGFR) and bevacizumab (anti- VEGF) in solid tumors has, however, been less dramatic. A reason for this may be the limited penetration of antibodies into the tumor but also an under appreciation of potential effector function. To improve upon existing therapies recombinant antibodies which have been optimized to elicit immune effector function (e.g. ADCC) are now emerging as a major new class of drugs [40]. With this in mind it is important to assess all potential mechanisms of killing for any novel drug. The identification of novel cancer targets will help to broaden the number of patients for which antibody-based immunotherapy may be effective. The demonstration of cathepsin S targeted ADCC efficacy opens up the possibility of targeting other enzymes (such as MMP's, ADAMs) in a similar way. Antibodies such as Fsn0503h, combining immune effector function with the ability to specifically block protease activity as demonstrated in our previous published data [23], may have significant clinical utilityfor the treatment of cancer.

## Conclusions

In summary, we demonstrate expression of cathepsin S on the surface of pancreatic and colon carcinoma cells and reveal how cell surface associated cathepsin S can be targeted to mediate ADCC by a fully humanized anti-cathepsin S antibody, Fsn0503h. This is the first example of a protease or surface enzyme being targeted to elicit ADCC. This data highlights the potential to target cell surface associated enzymes as therapeutic targets using antibodies capable of eliciting ADCC in tumor cells.

## Materials and Methods

### Cancer cell lines

The human cancer cell lines (Colo205, LoVo, BxPC-3, Panc-1 and Aspc-1) were purchased from American Type Culture Collection (ATCC, Rockville, MD). The colon carcinoma cell lines, Colo205 and LoVo, were cultured in RPMI 1640 medium (Sigma Aldrich, UK) and F-12K medium (Invitrogen, UK) respectively, supplemented with 10% foetal calf serum (FCS) (PAA, Laboratories, Somerset, UK) and L-glutamine (10 nmol/L) (Invitrogen, UK). BxPC-3, Aspc-1 and Panc-1 (pancreatic carcinoma) were cultured in RPMI 1640 medium (Invitrogen, UK) supplemented with 10% foetal calf serum (FCS) (PAA, Laboratories, Somerset, UK) and L-glutamine (10 nmol/L) (Invitrogen, UK). All cultures were maintained in a humidified environment at 37°C with 5% CO_2_.

### Immunohistochemistry (IHC) staining

Tissue microarray sections, containing biopsy cores taken from colorectal cancer patient specimens, were stained for cathepsin S as previously described [21] using an automated IHC platform (Bond Max™, Leica Microsystems, Newcastle, UK). Mouse anti-cathepsin S antibody Fsn0503 (Fusion Antibodies Ltd.) was used at 4 ug/mL. A polymer-based detection system (Refine cat#DS9800) was used with 3',3-Diaminobenzidine (DAB) as the chromogen.

### Western blotting and Flow cytometry

To prepare whole cell lysates of Colo205, LoVo, BxPC-3, Aspc-1 and Panc-1, cell pellets were washed in PBS and lysed using standard RIPA buffer supplemented with a Calbiochem^® ^protease inhibitor cocktail set III (1:50 dilution of lysis buffer) (Merck Chemicals Ltd., UK) as previously described [22]. Samples were incubated for 30 minutes on ice prior to a 20 minute centrifugation at 13,000 rpm. Protein concentration was determined by BCA assay (Thermo Scientific, UK). Denatured samples were analysed by SDS-PAGE on 12% (w/v) polyacrylamide gels. Gels were transferred by semi-dry blotting onto nitrocellulose membrane, blocked with 3% dried milk before probing with 0.4 μg mouse anti-Cathepsin S antibody in 15 mL PBS overnight 4°C. Following washes in phosphate-buffered saline tween (PBS-T), membranes were probed with goat anti-mouse-HRP (1:3000) (Bio-Rad Laboratories Ltd., UK) for 1 hour at room temperature. After a series of further washes with PBS-T, membranes were developed using Chemiluminescent Substrate (West Pico Chemiluminescent Substrate, Pierce) for 5 minutes.

The surface expression of cathepsin S on Colo205, LoVo, BxPC-3, Aspc-1 and Panc-1 cell lines was examined and quantified by flow cytometry. Briefly, cells (0.5 × 10^6^) were washed with cold Cell Wash (BD Biosciences, UK) and incubated with 0.5μg of polyclonal goat anti-cathepsin S antibody (R&D Systems, UK) in PBS for 1.5 hour on ice. The cells were then washed with cold Cell Wash and incubated with R-PE-conjugated donkey anti-goat antibody (1:50) (Abcam, UK) in PBS for 1 hour on ice. The cells were washed with cold PBS and stored at 4°C in the dark prior to analysis on the FACSCanto (BD Biosciences, UK). Target quantification was performed using QuantiBRITE PE beads (BD Biosciences, UK). Briefly, QuantiBRITE PE beads were run on the BD FACSCanto using the same parameters for fluorescence as used for the analysis of the cell lines. Geometric means were displayed in the statistic view (BD FACS Diva Software v6.1) and the calculation of Antibody Bound per Cell (ABC) values was carried out according to the manufacturer's protocol.

### Confocal microscopy

To assess localized cathepsin S expression Colo205, LoVo, BxPC-3, Aspc-1 and Panc-1 cells were grown on coverslips at 20,0000 cells/well in a humidified environment at 37°C with 5% CO_2 _overnight. The cells were then rinsed with Cell Wash (BD Biosciences, UK) and fixed with 4% paraformaldehyde (PFA) for 15 minutes at room temperature. After fixing, the cells were washed with PBS and non-specific sites were quenched with a blocking buffer (PBS containing 10% goat serum) for 2 hours. The cells were then incubated with 32 μg of mouse anti-cathepsin S antibody Fsn0503 or mouse isotype antibody control (Fusion Antibodies Ltd., UK) for 1 hour at 37°C. After extensive rinsing with PBS, the cells were incubated with 10 μg/mL goat anti-mouse AlexFlour 488-conjugated secondary antibody (Invitrogen, UK) in blocking buffer for 1 hour in the dark. Labelling of actin filaments was performed with Molecular Probes^® ^rhodamine phalloidin (1:150) (Invitrogen, UK). After extensive washing, the coverslips were mounted upside-down on glass microscope slides using Vectashield^® ^media (Vector Laboratories Inc., Burlingame, CA, USA) and viewed on laser confocal microscope.

### Humanization of the murine antibody

mRNA was extracted from 10^6 ^cells of Fsn0503 using STAT60 reagent. RT-PCR was performed using an oligo(dT) primer and reverse transcriptase, followed by PCR with murine specific IgG degenerate primers. The PCR products were cloned into the pCR2.1 vector (Invitrogen) for sequencing analysis. The vector was transformed into TOP10 cells and positive clones identified by PCR. The plasmid clones were mini-prepped and sequenced by the dideoxy-chain termination method on an ABI 3130xl genetic analyser. A consensus sequence for the light chain and heavy chain variable domains was determined from a minimum of 5 clones.

A Composite Human Antibody™ version of the Fsn0503 antibody, Fsn0503h, was designed from multiple human antibody sequences, with CDRs (complementarity Determining Regions) identified using Kabat and Chothia definitions (REF). The composite sequence was screened for T cell epitopes binding to human MHC Class II alleles (Antitope). The Fsn0503h V_H _and V_L _sequences were synthetically constructed and cloned into an expression vector containing human IgG1 isotype constant domains. Human IgG1 isotype was selected as the optimum isotype for ADCC activity in humans. NS01 cells were stably transfected via electroporation with both heavy chain and light chain expression vectors. Electroporated cells were distributed into 5 × 96 well plates and selected with 200 nM MTX (methotrexate). Wells containing methotrexate resistant colonies were sampled and tested for IgG expression levels, and the best expressing line was selected and frozen down (Antitope). Fsn0503h was then further selected by two rounds of limiting dilution in 96 well plates in the presence of 200 nM MTX. Fsn0503h cells were cultured in Dulbeccos Minimum Essential medium (Invitrogen, Paisley, UK) supplemented with 10% Ultra Low IgG serum (Invitrogen, Paisley, UK), 1% Penicillin Streptomycin (PAA laboratories, Austria), and 200 nM methotrexate (Sigma, Poole, UK). Cells were cultured in T-flasks (Greiner, GmbH). Cells were routinely passaged twice a week at ratios between 1:3 and 1:6 according to the cell density. To express large quantities of antibodycells were seeded in T-175 flasks at a seeding density of 3.0-5.0 × 10^4 ^cells/cm^2 ^in 30 mL of complete DMEM. Cells were incubated at 37°C 6% CO_2 _for 11 days prior to harvesting of culture medium by centrifugation at 5,000 g for 40 minutes. Antibody was purified on a protein A column (GE Healthcare, UK) using AKTA prime chromatography system (GE Healthcare, UK).

### Pharmacokinetic analysis

A PK study was performed to assess the stability and half-life of Fsn0503h in serum. All animal experiments were carried out in accordance with the Animal (Scientific Procedures) Act 1986 and conformed to current UK Co-ordinating Committee on Cancer Research guidelines. Eight week old male and female Sprague-Dawley (SD) rats (three male; three female), were housed in a temperature and humidity-controlled room for the duration of the study. Blood samples (pre-bleed) were taken from each animal before treatment with Fsn0503h. Each animal was administrated with 10 mg/kg Fsn0503h by intravenous (i.v.) tail injection. Blood samples (0.5 - 1 mL) were collected in heparinised tubes at selected time points following administration of Fsn0503h: 1, 4, 24, 48 hours, then once a week (7, 21, 28, 35, 42 days). Samples were centrifuged following collection, and serum was stored at -20°C until analysis by ELISA. Briefly, recombinant human cathepsin S was coated onto a 96-well plate (40 nM) and incubated with varying dilutions of serum or drug standards for 1 hour at room temperature. After rinsing with PBS-T secondary antibody (rat anti-human horseradish peroxidase-conjugated antibody) was added to each well. Plates were incubated for 1 hour at room temperature, washed with PBS-T and then incubated with TMB for 5 minutes at room temperature. The reaction was stopped by the addition of 500 mmol/L HCL, and absorbance was read at 450 nm.

### Cytotoxicity assays

ADCC was determined by conducting a standard CytoTox 96^® ^Non-Radioactive Cytotoxicity LDH-Release Assay purchased from Promega. The LoVo colorectal cancer cell line and BxPC3 pancreatic cancer cell line were selected for analysis. The LoVo and BxPC-3 cells were seeded at 7000 cells/well. Human peripheral blood mononuclear cells (PBMCs) obtained from healthy donors were used as effectors cells following IL-2 stimulation (100 U/mL). Both the target (T) and effector (E) cells were resuspended in 5% FCS RPMI medium with 15 mM HEPES and incubated in triplicate on 96-well microtiter plates with various concentrations of Fsn0503h (from 6670 to 833.75 nM) with or without CD16 neutralizing antibody (6670 nM) at an E:T cell ratio of 40:1.

### Statistical analysis

Statistical analysis was performed on experimental results using the student t test of variance. All *in vitro*/*ex vivo *experiments were repeated a minimum of three times and datais expressed as means ± SD.

## Competing interests

HFK, RJB, DK, JAG, DD, TJJ, AM, CW, TB, JJ, KLL, PS, CM, REB, BG, JL, IS, NG are (or were) employees of Fusion Antibodies Ltd. NC, AK, CJS and JAJ are consultants for Fusion Antibodies Ltd.

## Authors' contributions

HFK, DK, JAG and REB performed the IHC staining and western blotting. HFK, DK and AK performed the flow cytometry experiments. HFK and TJJ performed the confocal microscopy studies. HFK, RJB, CW, TB, JJ, KLL, PS, CM, BG, JL, IS, NG were responsible for humanization and production of Fsn0503. HFK and JAG performed the *in vivo *pharmacokinetic analysis. HFK, DD and AM performed the cytotoxicity assays. HFK, RJB, AK, JAJ and SAO drafted the manuscript. HFK, RJB, NC, CJS, JAJ and SAO participated in the initial conception, design and supervision of the study. All authors read and approved the final manuscript.
